# The National Medical Union (Official)

**Published:** 1914-05-02

**Authors:** 


### THE NATIONAL MEDICAL UNION (Official).

Offices: 346 Strand. Tel.: City 8444.

### THE CONSTITUTION COMMITTEE'S RECOMMENDATIONS.

A meeting of the Constitution Committee was held at the offices of the Union on Wednesday, April 22, Dr. Oppenheimer in the chair.

The committee decided to recommend that the National Medical Union should apply for registration under the Companies Act, 1867, as by so doing the Union would obtain a corporate existence and the liability of members would be limited.

### The Permanent Council.

It was considered that the first practical step was to proceed with the election of the permanent office-bearers and Council. In the case of the Council this should, of course, be on a representative basis, each member of Council representing a definite number of subscribers. It was proposed that the permanent officers should include two honorary presidents, not less than three honorary vice-presidents, secretaries, treasurers, and Council, with committees—executive, organising, finance, parliamentary, and press. The committee accordingly recommended that immediately after the general meeting, at which the honorary officers would be elected, the local Associations should themselves conduct an election for the membership of the first permanent Council. It was considered that one member of Council should be allowed for every Association of twenty-five subscribers and under, with an additional member for every twenty additional subscribers, the minimum membership entitling any Society to elect a member of Council being fixed at ten. Associations having under ten members would elect their representatives in common with the unattached members of the Union through the central office. This scheme, it was thought, would enable the Council of the Union to carry on its business without reaching an unwieldy size during its first year of office.

### The Permanent Committees.

It was agreed that the chairman of Council, together with the secretaries, should be *ex-officio* members of all committees, and that the following permanent committees should form part of the constitution:—

(1) An executive committee, consisting of twelve members, three *ex officio*, nine elected, the duties of this committee being to carry out decisions of the Council and in emergencies to exercise all the functions vested in the Council.

(2) An organising committee of twelve members, three *ex officio*, nine elected.

(3) A finance committee of eight members, five *ex officio*, the hon. treasurers being in this case *ex-officio* members.

(4) A parliamentary committee of eight members, three *ex officio*, five elected.

(5) A press committee of six members, three *ex officio* and three elected.

It was thought that the further steps in connection with the constitution would be best taken by the permanent Council when elected. These include the drawing up of a memorandum of association. This, it was specially pointed out, should be drafted so widely as to include every possible power which the Union might wish to exercise. Articles of association and bye-laws would also have to be prepared.

### The Question of a Business Manager.

Some discussion took place on the advisability or necessity of obtaining the services of a whole or part-time salaried business manager. The Hon. Secretaries explained that they themselves intended that their connection with the Union in any capacity should remain a purely honorary one, but that, if the Union was to receive adequate attention as a large organisation on business lines, some such provision as was suggested would have to be contemplated. The Chairman (and Treasurer) agreed with the suggestion, but pointed out that the practicability of the proposal depended upon funds being available.

[Further letters on Non-Panel Men and the "Medical Directory" appear on pages 133, 134.—Ed. The Hospital.]

### The Unallotted Medical Benefit Surplus.

The Insurance Committee for the County of London occupied nearly the whole time of their meeting on April 23 in discussing the problem of how to dispose of the sum of money, stated to be £200,000, which has accumulated under the National Insurance Act, and which represents largely contributions towards medical benefit of those insured persons in London who have preferred to be attended at their private expense by their own doctor rather than choose one from the panel.

It will be remembered that the Committee had twice taken Counsel's opinion at the expense of the Insurance funds, and on both occasions they had been advised that they could not legally distribute this money among panel doctors without rendering themselves personally liable to refund it. Counsel further expressed the opinion that any attempt to get round the situation by allocating patients either by number or name would be "in the nature of a dodge."

Nevertheless, the London Insurance Committee decided, although it is true by only the small majority of four, to continue the policy of kicking against the pricks, and the amendment finally carried was:—

"That, having considered the opinion of Counsel as to the distribution of the moneys in the panel fund for the year ending January 11, the Committee consider it their duty to prepare a scheme for the distribution of the said fund and to submit such scheme to the Insurance Commissioners for approval; and that the Medical Benefit Sub-Committee be instructed to prepare and submit such scheme to the next meeting of the Committee."

The position is now a peculiar one, and above and beyond all precedent. Here we have a public body who, as trustees of large sums of public money, have twice taken Counsel's opinion on an intricate point involving a sum which throughout the country amounts to a million pounds, consider it "their duty" to flout legal advice and proceed to instruct a sub-committee to draw up a scheme disposing of the money in a way which they have been advised is illegal.

The Committee's Responsibility and the Law.

The responsibility of the Committee is plain and will be dangerous to gamble with. It is merely a question of law. Much was said during the past year about the Insurance Act being now "the law of the land," and by some of those in the London Insurance Committee who are now so obstinately bent on ignoring a sane, deliberate, and well-considered interpretation of that law. The Insurance Commissioners cannot help them to do so, even if, as it is alleged, they are anxious to do so, for they have no power to legislate or alter the letter of the Act, and it is difficult to see how a sub-committee will undo the tangle, although it is to be noted that the above amendment, while providing for the preparation of a scheme "*for the distribution*" of the moneys, takes no count of the panel doctors, nor does it specifically suggest that they shall be the recipients of the surplus funds. About them, at any rate, the law is very explicit.

Another deduction, which, in spite of much equivocation and suppression of facts, may now fairly be drawn from the situation is that since a surplus of £200,000 of unallotted medical benefit has accrued in one year in the County of London, there must consequently be very nearly 600,000 persons, or one-third of the insured persons, who have taken their course and have declined to avail themselves of the so-called "benefit" of the panel doctor.

The Non-Panel Point of View.

This is extremely satisfactory from the point of view of the non-panel majority of the medical profession, and squares with the knowledge that quite **three-fourths of the medical** profession in London continue to abstain from panel service.

But it will be very surprising if the large number of insured persons who prefer their own doctor, now that they clearly understand the financial situation in London, will continue contributing towards what they have been hitherto told is their "medical benefit," and they will be less disposed to do so when they realise that their premiums for that benefit (which is denied them) go to swell a fund devoted to some altogether different object.

The difficulties of the London Insurance Committee have been entirely of their own making. Some of its members have apparently never realised that its function is to look after the interests of insured persons and administer its trust in accordance with the Act, while others spend their time in the vain pursuit of endeavouring to bring about that which they would, but which is not and cannot be.

The principle of National Insurance is admirable only in so far as those who would uphold it recognise that the interests of insured persons must never be made subservient to the "faculties" either of insurance or panel medicine.

### Lay Control of Panel Practice.

The conviction that the overwhelming predominance of lay representatives upon Insurance Committees would before long demonstrate the extent to which panel doctors have sacrificed their professional freedom and personal independence by accepting service under prevailing conditions has been strikingly illustrated by recent transactions of certain local Insurance Committees.

In Gloucester a panel practitioner, against whom it was alleged in Committee that he had failed to give proper attention to an insured person, and that he had given certain benefit certificates without seeing the person concerned, was removed from the panel, notwithstanding that more than eight hundred of his panel patients had petitioned against this action on the part of the Committee.

In Liverpool another panel doctor was similarly cashiered because it was charged against him that *on one occasion* during 1913 he could not be found at the address at which he had undertaken to give treatment and had provided no deputy.

But the most remarkable case is that of a panel practitioner in Hampshire, whose alleged offence was that he deliberately refused to see a man "outside his surgery hours"! For this he was informed by the Insurance Committee that a repetition of the offence would result in his removal from the panel.

When, in addition to such incidents as the above, we read that a larger and what should be a much more enlightened Insurance Committee have with due official gravity recently laid down that "the cutting of tonsils" (*sic*) and "the syringing of ears" must for the future be considered as compulsory panel tasks, it is surprising how any section of the profession can continue to barter their liberty, risk the loss of their means of subsistence at the caprice of a lay Committee, and compromise the good name of their profession in exchange for a service which was justly condemned as "derogatory and unworkable."

Meeting of Executive Committee.

A meeting of the Executive Committee is being held on Saturday, May 2, to fix the date of the first General Meeting of the Union, to appoint a Parliamentary Committee, and to consider certain further matters of policy referred to this committee at the last meeting of Council.

